# Associations of childhood trauma with clinical features and inflammatory cytokines in adolescents with first-episode and recurrent major depressive disorder

**DOI:** 10.3389/fimmu.2026.1787595

**Published:** 2026-03-03

**Authors:** Xi Zhang, Lewei Liu, Ruitong Li, Yun Zhang, Haojie Fan, Mingru Hao, Feng Geng, Daming Mo, Lei Xia, Huanzhong Liu

**Affiliations:** 1 Department of Psychiatry, The Fourth Affiliated Hospital of Anhui Medical University, Hefei, Anhui, China; 2 Department of Psychiatry, School of Mental Health and Psychological Sciences, Anhui Medical University, Hefei, Anhui, China; 3 Department of Psychology and Sleep Medicine, The Second Affiliated Hospital of Anhui Medical University, Hefei, Anhui, China; 4 Affiliated Psychological Hospital of Anhui Medical University, Hefei, Anhui, China

**Keywords:** adolescent, alexithymia, childhood trauma, depressive disorder, inflammatory cytokines, suicidal ideation

## Abstract

**Background:**

Childhood trauma is a significant risk factor for adolescents with major depressive disorder (MDD), but its associations with clinical features and inflammatory cytokines remain unclear across illness stages. This study aimed to investigate these associations by comparing adolescents with first-episode and recurrent MDD.

**Methods:**

We recruited 170 adolescents with MDD and 76 healthy controls (HCs) between January 2021 and December 2022. The Childhood Trauma Questionnaire (CTQ), the Center for the Epidemiological Studies Depression Scale (CES-D), the Positive and Negative Suicidal Ideation Scale (PANSI), and the 20-item Toronto Alexithymia Scale (TAS-20) were used to assess childhood trauma, severity of depression, suicidal ideation, and alexithymia. Plasma levels of interleukin (IL)-1β, IL-6, IL-8, IL-10, IL-17A, and tumor necrosis factor-alpha (TNF-α) were determined by electrochemiluminescence.

**Results:**

The detection rate of childhood trauma was significantly higher in the MDD group than in HCs (80.59% vs. 31.58%). Patients also had significantly higher scores on all clinical scales and elevated levels of IL-1β, IL-6, IL-10, IL-17A, and TNF-α (all *P* < 0.001), except for IL-8 (*P* = 0.543). Correlation analysis revealed that in first-episode patients, CTQ scores were positively linked to scores of CES-D, PANSI, and TAS-20 and levels of IL-1β, IL-6, IL-10, and IL-17A. However, in the total sample and recurrent patients, CTQ scores correlated solely with clinical features. Further multivariate linear stepwise regression analysis revealed that in first-episode patients, CTQ scores were independently associated with CES-D scores (*β* = 0.396, *t* = 3.688, *P* < 0.001), PANSI scores (*β* = 0.519, *t* = 5.190, *P* < 0.001), TAS-20 scores (*β* = 0.454, *t* = 4.355, *P* < 0.001), and levels of IL-1β (*β* = 0.264, *t* = 2.336, *P* = 0.022), IL-6 (*β* = 0.228, *t* = 2.002, *P* = 0.049), IL-10 (*β* = 0.253, *t* = 2.233, *P* = 0.029), and IL-17A (*β* = 0.251, *t* = 2.215, *P* = 0.030).

**Conclusion:**

Childhood trauma is common in adolescents with MDD and associated with more severity of depression, suicidal ideation, and alexithymia. Its link to inflammatory cytokines is exclusively observed in first-episode patients, suggesting trauma-related immune activation may be particularly important in initial onset.

## 1 Introduction

Major depressive disorder (MDD) is a highly disabling mental disorder marked by core symptoms such as depressed mood, loss of interest, and anhedonia, imposing a heavy global burden of disease (1, 2). According to epidemiological estimates, the percentage of Chinese children and adolescents with depressive disorders is around 2.0% (3). Given that adolescence is a phase of crucial brain development and cognitive maturation, depression in this stage tends to be associated with more severe symptoms, a higher risk of recurrence, and a poorer long-term prognosis (4). Therefore, investigating the underlying mechanisms is of paramount importance for both clinical practice and public health.

Childhood trauma is widely recognized as a pivotal environmental risk factor in MDD. Research indicated that experiencing childhood trauma early in life may markedly increase the risk of developing depressive disorders in adolescents (5). Supporting this, a cohort study by Houtepen et al. (6) revealed that children and adolescents with a history of childhood trauma had 2.4 times the risk of developing depression in the future compared to their peers without such trauma. Furthermore, childhood trauma exerted a substantial impact on the psychopathological symptoms of adolescents with MDD, such as triggering more intense suicidal ideation and more pronounced alexithymia (7, 8). The studies by Kayan et al. and Lee et al.also indicated that childhood trauma is closely associated with suicidal ideation and behavior in depressed adolescents (9, 10). And substantial evidence supported that childhood trauma serves as a significant risk factor for difficulties in emotion recognition (i.e., alexithymia) in adolescents (11–13).

The long-term influence of childhood trauma is not confined to the psychological realm with growing evidence suggestingit may also lead to dysregulation of the inflammatory system (14–18). Specifically, a clinical study in Brazil observed that among adolescents with MDD, childhood trauma was closely associated with increased levels of inflammatory cytokines, including IL-6 and IL-10 (14). Similarly, a recent meta-analysis has demonstrated an overall association between childhood trauma and increased levels of inflammatory cytokines (e.g., IL-6 and TNF-α) in individuals with depression (16). However, the association remains debated, as some studies have reported no significant direct link between the two. For instance, Oliveira et al. (19) reported that while depressive patients had elevated levels of inflammatory cytokines (e.g., IL-1β, IL-6, IL-17A, and TNF-α) compared to healthy controls, they failed to find a significant link between childhood trauma history and these cytokine levels among the patients. The inconsistent results are likely attributable to methodological heterogeneity, including differences in participant characteristics and insufficient adjustment for crucial confounders, notably pharmacotherapy. It is noteworthy that inflammatory dysregulation itself may be linked to a spectrum of adverse outcomes, including the development of depression, increased suicide risk, and cognitive decline (20–22). Therefore, investigating the potential associations between childhood trauma and clinical features as well as inflammatory cytokines in adolescents with MDD is of significant importance.

Based on the above, this study aimed to systematically investigate (1) the detection rate of childhood trauma in adolescents with MDD; (2) the differences of childhood trauma, clinical features, and the levels of inflammatory cytokines between adolescents with MDD and healthy controls (HCs); and (3) whether the associations between childhood trauma and the aforementioned indicators exhibit disease-stage specificity, by distinguishing illness stages (first-episode vs. recurrent) and controlling for key confounding factors such as medication treatment.

## 2 Materials and methods

### 2.1 Study design and participants

We conducted this cross-sectional study from January 2021 to December 2022. The participant cohort comprised 170 adolescent patients diagnosed with MDD, who were recruited from two clinical sites: the Fourth Affiliated Hospital and the Affiliated Psychological Hospital of Anhui Medical University. Inclusion criteria were: (1) aged 13–18 years, regardless of sex; (2) diagnosed with MDD based on the Diagnostic and Statistical Manual of Mental Disorders, fifth edition (DSM-5) criteria; and (3) provision of written informed assent and consent from both the adolescent and their parent/guardian after a full explanation of the study procedures, including clinical assessments and blood sampling. Exclusion criteria were: (1) a comorbid diagnosis of a psychiatric disorder such as bipolar disorder, schizophrenia, intellectual disability, and et al; (2) a serious concurrent medical condition, such as active infection, neurological disease, or other severe physical illness; (3) taking non-steroidal anti-inflammatory drugs (NSAIDs), corticosteroids, or immunomodulators within the past month or at the time of the study. Concurrently, 76 HCs were recruited from Hefei and surrounding areas. Inclusion criteria were: (1) aged 13–18 years, regardless of sex; (2) understood the study objectives and voluntarily agreed to participate in the assessments and blood sample collection. Exclusion criteria were: (1) a personal or family history of any psychiatric disorder; (2) a serious concurrent medical condition, such as active infection, neurological disease, or other severe physical illness; (3) taking non-steroidal anti-inflammatory drugs (NSAIDs), corticosteroids, or immunomodulators within the past month or at the time of the study. Before the study commenced, detailed information on the purpose and procedures was given to all participants and guardians, from whom written informed consent was then obtained. The Ethics Committee of Chaohu Hospital, Anhui Medical University granted ethical approval for this study (approval number: 202009-KYXM-04).

### 2.2 Measuring instruments

#### 2.2.1 Demographic characteristics

A study-specific questionnaire was used to collect demographic characteristics from all participants, which included age (years), sex (male/female), body mass index (BMI) (kg/m^2^), first-episode status (yes/no), age of onset (years), duration of illness (months), and use of antidepressants (none/SSRIs/others).

#### 2.2.2 Childhood trauma

We employed the 28-item Childhood Trauma Questionnaire (CTQ) to assess exposure to childhood trauma (23). This self-report instrument quantifies the severity of childhood adversity. Its five subscales measure emotional abuse, physical abuse, sexual abuse, emotional neglect, and physical neglect. Items (5–7 per subscale) are rated on a 5-point Likert scale from 1 (Never true) to 5 (Very often true). This yields subscale scores of 5–25 and a total score of 25-125, with higher totals reflecting more severe childhood trauma (24). The Chinese version of the CTQ has demonstrated robust psychometric properties, having been extensively validated and widely employed in adolescent cohorts across China (25).

#### 2.2.3 Severity of depression

We employed the 20-item Center for the Epidemiological Studies Depression Scale (CES-D) to measure severity of depression (26). The scale assesses the frequency of depressive symptoms experienced over the preceding week. All items are rated on a 4-point Likert scale. The total score, which ranges from 0 to 60, reflects the severity of depression, with higher scores representing more severe. The CES-D is a reliable and valid instrument for assessing depression in Chinese adolescents (27).

#### 2.2.4 Suicidal ideation

We employed the 14-item Positive and Negative Suicidal Ideation Scale (PANSI) to assess suicidal ideation (28). This self-report tool is specifically designed to measure the severity of suicidal ideation. All items are rated on a 5-point Likert scale. The total score, which ranges from 14 to 70, reflects the severity of suicidal ideation, with higher scores representing a greater severity. The Chinese version of the PANSI used in this study has been widely utilized and validated among adolescents with depression in China (29).

#### 2.2.5 Alexithymia

We employed the 20-item Toronto Alexithymia Scale (TAS-20) to assess alexithymia (30). This self-report instrument is a widely recognized and well-validated tool for measuring the alexithymia. All items are rated on a 5-point Likert scale. The total score, which ranges from 20 to 100, reflects the severity of alexithymia, with higher scores representing a greater severity. The Chinese version of the TAS-20 demonstrates good reliability and validity and is considered appropriate for assessing alexithymia symptoms in Chinese adolescent populations (31).

### 2.3 Measurement of inflammatory cytokines

After an overnight fast, venous blood samples were collected from all participants the next morning into ethylenediaminetetraacetic acid (EDTA) anticoagulant tubes. The samples were immediately centrifuged at 4 °C (3000 rpm for 15 minutes). The obtained plasma supernatant was aliquoted and stored at -80 °C until analysis. Plasma levels of inflammatory cytokines, including interleukin (IL)-1β, IL-6, IL-8, IL-10, IL-17A, and tumor necrosis factor-alpha (TNF-α), were analyzed using a high-sensitivity multiplex electrochemiluminescence assay.

### 2.4 Statistical analysis

All statistical analyses were performed using SPSS software (version 23.0). Before conducting the data, inflammatory cytokine levels (IL-1β, IL-6, IL-8, IL-10, IL-17A, TNF-α) were log10-transformed to normalize their skewed distributions. For continuous variables, data are presented as the mean ± standard deviation (SD) or as the median (quartiles) [M (P25, P75)], based on their distribution. Depending on whether they met normality assumptions (assessed via the Kolmogorov-Smirnov test), continuous variables were compared between groups with either the independent samples t-test (for normally distributed data) or the Mann-Whitney U test (for non-normally distributed data). Categorical variables are expressed as frequency and percentage [n (%)] and were compared using the Chi-square (*χ*²) test. In this study, correlation analysis was performed. Depending on data characteristics, correlations were assessed with Spearman (for categorical or non-normal continuous variables) or Pearson (for normally distributed continuous variables). Bonferroni corrections were applied to these analyses to adjust for multiple tests. Subsequently, based on the results of correlation analysis, the associations between childhood trauma and both clinical features and inflammatory cytokines were predominantly observed in first-episode with MDD. Therefore, to further clarify the role of childhood trauma as an independent predictor in the early stage of the illness, we focused our analysis on the subgroup of first-episode patients with MDD (n=75). In this subgroup, the stepwise method was employed in the multivariate linear regression analysis. Variables that correlated significantly (*P* < 0.05) (**Supplementary Table 1**) in the prior analysis were entered as dependent variables, with the CTQ scores as the independent variable. The duration of illness and BMI were included as covariates in the model. Statistical significance was considered at P < 0.05 (bilateral).

## 3 Results

### 3.1 Demographic characteristics, clinical features, and levels of inflammatory cytokines

From January 2021 to December 2022, we recruited 170 adolescents with MDD. Among them, males accounted for 27.65% of the sample. The mean age was 15.44 ± 1.46 years, the mean age at onset was 13.69 ± 1.86 years, and the mean duration of illness was 21.40 ± 19.04 months. The detection rate of childhood trauma was 80.59% (n = 137) (**Tables 1**, **2**). Concurrently, 76 healthy adolescents without any psychiatric or physical diseases were recruited as controls. Among them, males accounted for 59.21%, the mean age was 15.11 ± 1.76 years, and the detection rate of childhood trauma was 31.58% (n = 24), which was significantly lower than that in the MDD group (*P* < 0.001) (**Table 1**). Regarding pharmacotherapy, 67.36% of the recurrent MDD were taking antidepressants. Among them, selective serotonin reuptake inhibitors (SSRIs) were the most common (53.68%), while other types of medications accounted for 13.68% (**Table 2**).

**Table 1:** Comparisons of demographic characteristics, clinical features, and levels of inflammatory cytokines between adolescents with MDD and HCs.

Variables	MDD (n = 170)	HCs(*n* = 76)	*t/Z/χ^2^*	*P*
Demographic characteristics				
Males, n (%)	47 (27.65)	45 (59.21)	22.349 ^b^	**<0.001**
age (years), mean (SD)	15.44 (1.46)	15.11 (1.76)	1.561	0.120
BMI (kg/m^2^), mean (SD)	21.11 (4.25)	21.16 (4.48)	-0.074	0.941
Clinical features				
CES-D scores, median (P25, P75)	40.00 (30.00, 47.00)	6.00 (2.25, 10.00)	11.765 ^a^	**<0.001**
PANSI scores, mean (SD)	49.02 (13.09)	20.25 (5.61)	24.132	**<0.001**
TAS-20 scores, mean (SD)	68.29 (8.72)	56.49 (12.63)	7.398	**<0.001**
Childhood trauma, n (%)			55.784 ^b^	**<0.001**
Yes	137 (80.59)	24 (31.58)		
No	33 (19.41)	52 (68.42)		
CTQ scores, mean (SD)	51.56 (12.87)	34.67 (6.34)	13.777	**<0.001**
Emotional abuse, median (P25, P75)	12.00 (8.00, 15.00)	6.00 (5.00, 7.00)	8.897 ^a^	**<0.001**
Physical abuse, median (P25, P75)	6.00 (5.00, 9.00)	5.00 (5.00, 5.00)	6.233 ^a^	**<0.001**
Sexual abuse, median (P25, P75)	5.00 (5.00, 5.00)	5.00 (5.00, 5.00)	3.378 ^a^	**0.001**
Emotional neglect, mean (SD)	16.03 (5.24)	9.55 (3.44)	11.490	**<0.001**
Physical neglect, mean (SD)	10.61 (3.38)	8.08 (3.06)	5.578	**<0.001**
Inflammatory cytokines				
Log IL-1β (ng/L), mean (SD)	-0.54 (0.42)	-0.73 (0.25)	4.394	**<0.001**
Log IL-6 (ng/L), mean (SD)	0.29 (0.28)	0.16 (0.19)	4.458	**<0.001**
Log IL-8 (ng/L), mean (SD)	0.27 (0.24)	0.25 (0.15)	0.609	0.543
Log IL-10 (ng/L), mean (SD)	-0.25 (0.33)	-0.32 (0.17)	2.115	**0.035**
Log IL-17A (ng/L), mean (SD)	0.38 (0.32)	0.31 (0.20)	1.974	**0.049**
Log TNF-α (ng/L), mean (SD)	0.09 (0.26)	-0.03 (0.13)	4.473	**<0.001**

BMI, body mass index; CES-D, center for the epidemiological studies depression scale; PANSI, positive and negative suicidal ideation scale; TAS-20, 20-item toronto alexithymia scale; CTQ, childhood trauma questionnaire; IL, interleukin; TNF, tumor necrosis factor; a, Mann-Whitney U test; b, Chi-square (*χ*²) test; Bolded *P* value: < 0.05.

**Table 2:** Comparisons of first-episode and recurrent adolescents with MDD.

Variables	Total sample (*n* = 170)	First-episode MDD (*n* = 75)	Recurrent MDD (*n* = 95)	*t/Z/χ^2^*	*P*
Demographic characteristics					
Males, n (%)	47 (27.65)	21 (28.00)	26 (27.37)	0.008 ^b^	0.927
age (years), mean (SD)	15.44 (1.46)	15.55 (1.34)	15.36 (1.55)	0.837	0.404
BMI (kg/m^2^), mean (SD)	21.11 (4.25)	20.88 (4.06)	21.30 (4.41)	-0.632	0.528
Age of onset (years), mean (SD)	13.69 (1.86)	13.59 (1.94)	13.78 (1.81)	-0.667	0.505
Duration of illness (months), median (P25, P75)	16.00 (11.00, 24.00)	16.00 (10.00, 24.00)	12.00 (16.00, 24.00)	-0.617 ^a^	0.537
Antidepressants				81.033 ^b^	**<0.001**
None, n (%)	106 (62.35)	75 (100.00)	31 (32.63)		
SSRIs, n (%)	51 (30.00)	0 (0.00)	51 (53.68)		
Others, n (%)	13 (7.65)	0 (0.00)	13 (13.68)		
Clinical features					
CES-D scores, mean (SD)	37.85 (12.25)	37.40 (12.89)	38.20 (11.78)	-0.422	0.674
PANSI scores, mean (SD)	49.02 (13.09)	49.31 (12.62)	48.80 (13.51)	0.250	0.803
TAS-20 scores, mean (SD)	68.29 (8.72)	68.43 (9.41)	68.19 (8.19)	0.176	0.861
Childhood trauma, n (%)				0.048 ^b^	0.827
Yes	137 (80.59)	61 (81.33)	76 (80.00)		
No	33 (19.41)	14 (18.67)	19 (20.00)		
CTQ scores, mean (SD)	51.56 (12.87)	51.43 (12.75)	51.66 (13.03)	-0.119	0.906
Emotional abuse, mean (SD)	11.89 (4.64)	12.03 (4.69)	11.79 (4.62)	0.330	0.741
Physical abuse, median (P25, P75)	6.00 (5.00, 9.00)	6.00 (5.00, 9.00)	6.00 (5.00, 8.50)	-0.300 ^a^	0.764
Sexual abuse, median (P25, P75)	5.00 (5.00, 5.00)	5.00 (5.00, 5.00)	5.00 (5.00, 5.00)	-1.777 ^a^	0.076
Emotional neglect, mean (SD)	16.03 (5.24)	15.45 (5.11)	16.48 (5.33)	-1.275	0.204
Physical neglect, mean (SD)	10.61 (3.38)	10.67 (3.62)	10.56 (3.19)	0.208	0.836
Inflammatory cytokines					
Log IL-1β (ng/L), mean (SD)	-0.54 (0.42)	-0.58 (0.36)	-0.51 (0.47)	-1.185	0.238
Log IL-6 (ng/L), mean (SD)	0.29 (0.28)	0.26 (0.23)	0.32 (0.32)	-1.179	0.240
Log IL-8 (ng/L), mean (SD)	0.27 (0.24)	0.24 (0.17)	0.30 (0.29)	-1.773	0.078
Log IL-10 (ng/L), mean (SD)	-0.25 (0.33)	-0.24 (0.25)	-0.26 (0.38)	0.412	0.681
Log IL-17A (ng/L), mean (SD)	0.38 (0.32)	0.36 (0.29)	0.39 (0.34)	-0.674	0.501
Log TNF-α (ng/L), mean (SD)	0.09 (0.26)	0.07 (0.20)	0.10 (0.30)	-0.688	0.492

BMI, body mass index; SSRIs, selective serotonin reuptake inhibitors; CES-D, center for the epidemiological studies depression scale; PANSI, positive and negative suicidal ideation scale; TAS-20, 20-item toronto alexithymia scale; CTQ, childhood trauma questionnaire; IL, interleukin; TNF, tumor necrosis factor; a, Mann-Whitney U test; b, Chi-square (*χ*²) test; Bolded P value: < 0.05.

**Figure 1:**
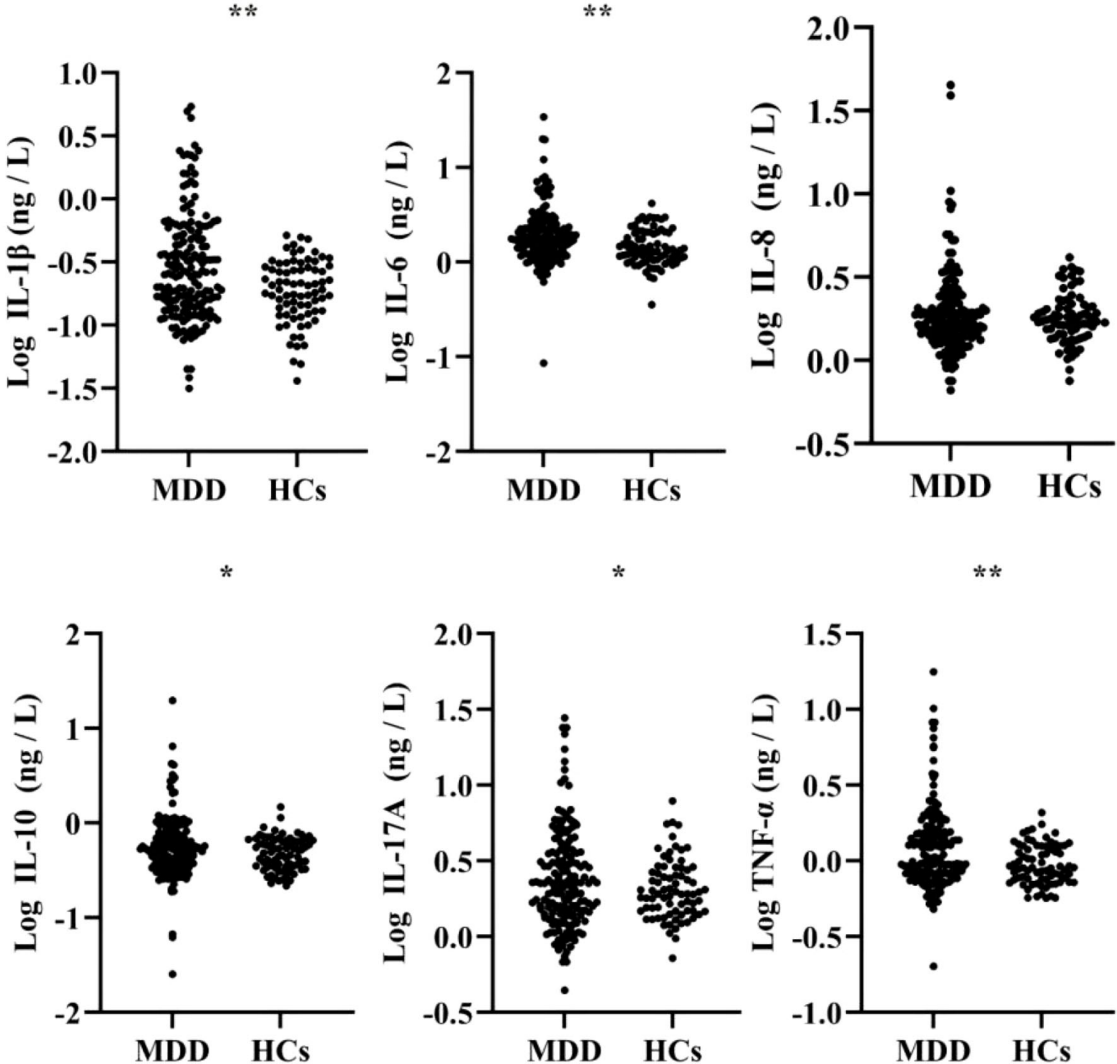
Comparisons of inflammatory cytokines between adolescents with MDD and HCs. *P < 0.05; **P < 0.001.

Compared with the HCs, adolescents with MDD had significantly higher CTQ scores (total and all subscales), CES-D scores, PANSI scores, TAS-20 scores, as well as plasma levels of IL-1β, IL-6, IL-10, IL-17A, and TNF-α (all *P* < 0.001), while no statistically significant difference was found in IL-8 levels between the two groups (*P* = 0.543) (**Table 1**, **Figure 1**). In addition, no statistically significant differences were observed in clinical features or levels of inflammatory cytokines between the first-episode and recurrent MDD (all *P* > 0.05) (**Table 2**).

### 3.2 The correlation between CTQ scores and demographic characteristics, clinical features, and inflammatory cytokines

To investigate the association between childhood trauma and various research variables, we conducted a correlation analysis. The results showed that CTQ scores were correlated with clinical features, but the correlation with levels of inflammatory cytokines exhibited clear stage specificity (**Table 3**).

**Table 3:** The correlation between CTQ scores and demographic characteristics, clinical features, and inflammatory cytokines.

Variables	Total sample (*n* = 170)		First-episode MDD (*n* = 75)		Recurrent MDD (*n* = 95)	
	*r*	*P*	*r*	*P*	*r*	*P*
Demographic characteristics						
Sex	-0.180	0.121	-0.180	0.121	-0.218	**0.034**
age (years)	-0.241	**0.002**	-0.194	0.095	-0.273	**0.008**
BMI (kg/m^2^)	-0.051	0.510	0.082	0.484	-0.146	0.158
Age of onset (years)	-0.132	0.085	0.036	0.758	-0.273	**0.007**
Duration of illness (months)	0.241	**0.037**	0.241	**0.037**	0.193	0.061
Antidepressants	-0.029	0.711	–	–	-0.064	0.538
Clinical features						
CES-D scores	0.539	<0.001*	0.396	<0.001*	0.661	<0.001*
PANSI scores	0.632	<0.001*	0.519	<0.001*	0.714	<0.001*
TAS-20 scores	0.352	<0.001*	0.454	<0.001*	0.264	**0.010**
Inflammatory cytokines						
Log IL-1β (ng/L)	0.078	0.313	0.264	**0.022**	-0.033	0.754
Log IL-6 (ng/L)	0.068	0.376	0.228	**0.049**	-0.019	0.854
Log IL-8 (ng/L)	0.018	0.818	0.096	0.414	-0.018	0.863
Log IL-10 (ng/L)	-0.042	0.586	0.253	**0.029**	-0.191	0.064
Log IL-17A (ng/L)	0.110	0.155	0.251	**0.030**	0.019	0.854
Log TNF-α (ng/L)	0.052	0.501	0.219	0.059	-0.033	0.752

BMI, body mass index; CES-D, center for the epidemiological studies depression scale; PANSI, positive and negative suicidal ideation scale; TAS-20, 20-item toronto alexithymia scale; CTQ, childhood trauma questionnaire; IL, interleukin; TNF, tumor necrosis factor. *P < 0.05/45 = 0.001 (Bonferroni correction). Bolded P value: < 0.05.

In the total sample (n = 170), CTQ scores were significantly positively correlated with the CES-D scores (r = 0.539, *P* < 0.001), PANSI scores (r = 0.632, *P* < 0.001), and TAS-20 scores (r = 0.352, *P* < 0.001); However, CTQ scores showed no significant correlation with plasma levels of any inflammatory cytokine measured, including IL-1β, IL-6, IL-8, IL-10, IL-17A, and TNF-α (all *P* > 0.05). After Bonferroni correction (α = 0.05/45 = 0.001), the correlations between CTQ scores with CES-D scores, PANSI scores, and TAS-20 scores remained significant.

**Figure 2:**
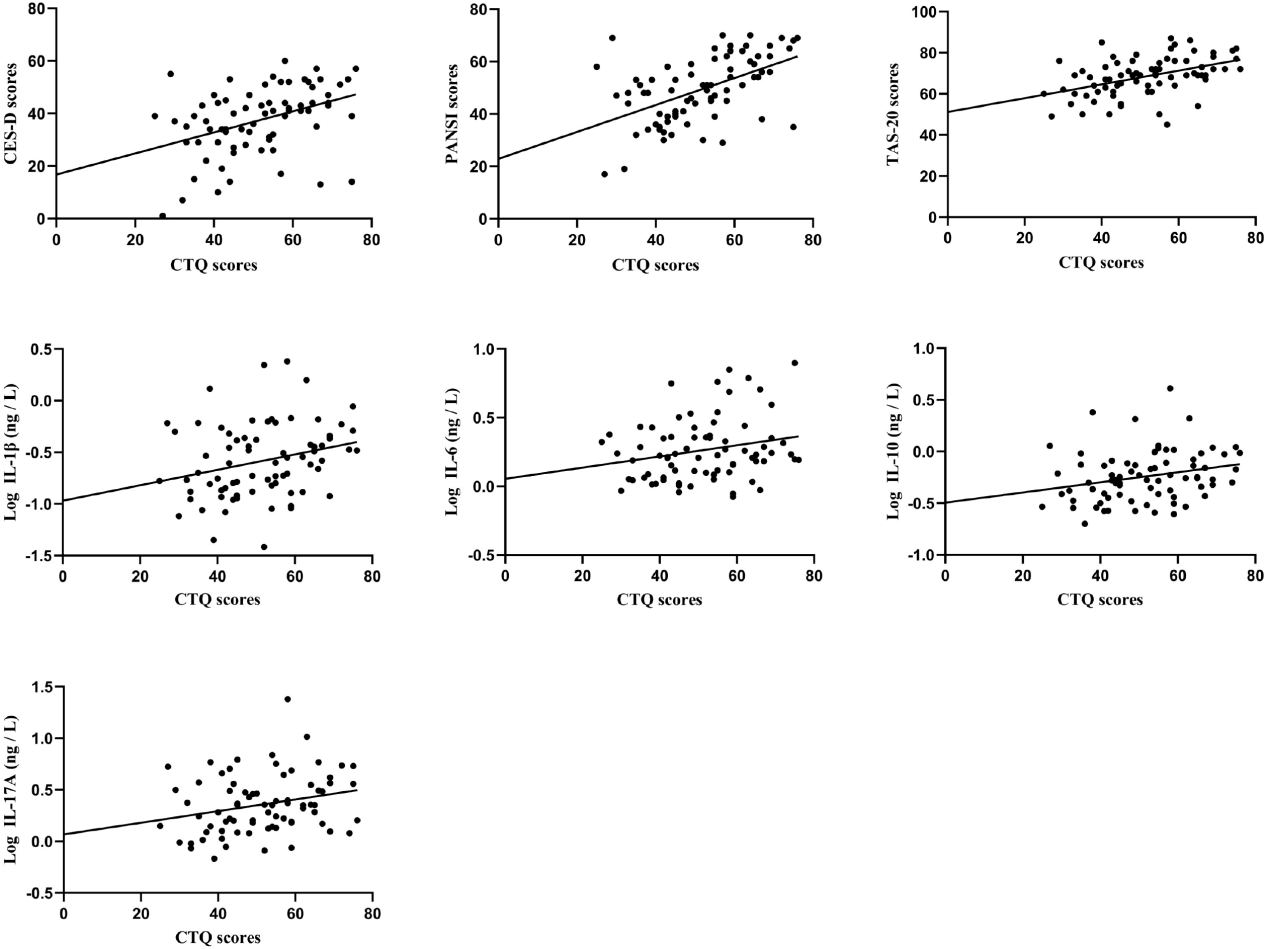
Correlation of CTQ scores with CES-D, PANSI, TAS-20, and levels of Log IL-1β, Log IL-6, Log IL-10, Log IL-17A.

After stratifying the samples by disease stage, key differences were observed in their associations. In first-episode MDD group (n = 75), CTQ scores were not only significantly positively correlated with CES-D scores (r = 0.396, *P* < 0.001), PANSI scores (r = 0.519, *P* < 0.001), and TAS-20 scores (r = 0.454, *P* < 0.001), but also with the levels of IL-1β (r = 0.264, *P* = 0.022), IL-6 (r = 0.228, *P* = 0.049), IL-10 (r = 0.253, *P* = 0.029) and IL-17A (r = 0.251, *P* = 0.030) (**Figure 2**). After Bonferroni correction (α = 0.05/45 = 0.001), the correlations between CTQ scores with CES-D scores, PANSI scores, and TAS-20 scores remained significant; all correlations with inflammatory cytokines were non-significant.

In contrast, among recurrent MDD group (n = 95), although CTQ scores were significantly positively correlated with CES-D scores (r = 0.661, *P* < 0.001), PANSI scores (r = 0.714, *P* < 0.001) and TAS-20 scores (r = 0.264, *P* = 0.010), they showed no significant correlation with any levels of inflammatory cytokines (all *P* > 0.05). After Bonferroni correction (α = 0.05/45 = 0.001), the correlations with CES-D and PANSI scores remained significant, whereas the correlation with TAS-20 scores was non-significant.

### 3.3 The correlation between inflammatory cytokines and clinical features

**Figure 3:**
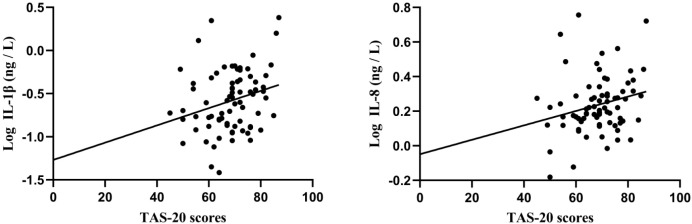
Correlation of TAS-20 scores with levels of Log IL-1β, Log IL-8.

We also conducted a correlation analysis between inflammatory cytokines and clinical features in first-episode and recurrent with MDD. The results show that, in first-episode with MDD, IL-1β (r = 0.261, *P* = 0.024) and IL-8 (r = 0.233, *P* = 0.044) showed significantly positive correlations with TAS-20 scores, whereas no other significant correlations were observed between inflammatory cytokines and clinical features. However, these correlations did not survive Bonferroni correction (α = 0.05/18 = 0.003). By contrast, all correlations between inflammatory cytokines and clinical features were non-significant in recurrent patients (all *P* > 0.05) (**Table 4**, **Figure 3**).

**Table 4:** The correlation between inflammatory cytokines and clinical features in first-episode and recurrent with MDD.

Variables	First-episode MDD (*n* = 75)			Recurrent MDD (*n* = 95)		
	CES-D scores *r* (*p*)	PANSI scores *r* (*p*)	TAS-20 scores *r* (*p*)	CES-D scores *r* (*p*)	PANSI scores *r* (*p*)	TAS-20 scores *r* (*p*)
Log IL-1β (ng/L)	0.052 (0.657)	0.053 (0.653)	0.261 (**0.024**)	0.098 (0.344)	0.022 (0.833)	0.042 (0.683)
Log IL-6 (ng/L)	-0.003 (0.978)	0.007 (0.953)	0.156 (0.181)	0.178 (0.084)	0.098 (0.343)	0.026 (0.805)
Log IL-8 (ng/L)	0.107 (0.362)	-0.070 (0.552)	0.233 (**0.044**)	0.022 (0.832)	-0.029 (0.782)	-0.061 (0.554)
Log IL-10 (ng/L)	0.058 (0.621)	0.095 (0.419)	0.177 (0.128)	-0.180 (0.081)	-0.172 (0.095)	0.068 (0.516)
Log IL-17A (ng/L)	0.003 (0.978)	0.023 (0.845)	0.205 (0.078)	0.152 (0.142)	0.096 (0.357)	0.073 (0.482)
Log TNF-α (ng/L)	0.016 (0.890)	-0.007 (0.954)	0.189 (0.105)	0.104 (0.318)	0.014 (0.894)	0.007 (0.947)

CES-D, center for the epidemiological studies depression scale; PANSI, positive and negative suicidal ideation scale; TAS-20, 20-item toronto alexithymia scale; CTQ, childhood trauma questionnaire; IL, interleukin; TNF, tumor necrosis factor. Bolded P value: < 0.05.

### 3.4 Independent factors associated with CTQ scores in adolescents with first-episode MDD

To further clarify the influence of childhood trauma in the early stage of the disease, we conducted a series of multivariate linear stepwise regression analyses in adolescents with first-episode MDD (n = 75), with CTQ scores as the independent variable and clinical scores or levels of inflammatory cytokines as the dependent variables. The results showed that in adolescents with first-episode MDD, CTQ scores were independently associated with CES-D scores (*β* = 0.396, *t* = 3.688, *P* < 0.001), PANSI scores (*β* = 0.519, *t* = 5.190, *P* < 0.001), TAS-20 scores (*β* = 0.454, *t* = 4.355, *P* < 0.001), and the levels of IL-1β (*β* = 0.264, *t* = 2.336, *P* = 0.022), IL-6 (*β* = 0.228, *t* = 2.002, *P* = 0.049), IL-10 (*β* = 0.253, *t* = 2.233, *P* = 0.029), and IL-17A (*β* = 0.251, *t* = 2.215, *P* = 0.030) (**Table 5**).

**Table 5:** Independent factors associated with CTQ scores by multivariate linear stepwise regression analysis.

Variables	*B*	*95% CI*	*SE*	*β*	*t*	*P*
CES-D scores	0.401	0.184~0.617	0.109	0.396	3.688	**<0.001**
PANSI scores	0.514	0.317~0.711	0.099	0.519	5.190	**<0.001**
TAS-20 scores	0.335	0.182~0.488	0.077	0.454	4.355	**<0.001**
Log IL-1β (ng/L)	0.007	0.001~0.014	0.003	0.264	2.336	**0.022**
Log IL-6 (ng/L)	0.004	0.001~0.008	0.002	0.228	2.002	**0.049**
Log IL-10 (ng/L)	0.005	0.001~0.009	0.002	0.253	2.233	**0.029**
Log IL-17A (ng/L)	0.006	0.001~0.011	0.003	0.251	2.215	**0.030**

CES-D, center for the epidemiological studies depression scale; PANSI, positive and negative suicidal ideation scale; TAS-20, 20-item toronto alexithymia scale; CTQ, childhood trauma questionnaire; IL, interleukin; Bolded P value: < 0.05.

## 4 Discussion

This study found that, compared to HCs, adolescents with MDD in our sample showed significantly higher childhood trauma burden, both in terms of detection rate (80.59%) and CTQ scores (total and across all subscales), which is consistent with the existing literature (32, 33). Early childhood trauma may increase an individual’ s vulnerability to depression through multiple pathways, such as compromising psychological resilience, disrupting metabolic processes, and impairing cognitive function (34, 35). However, it is not clear whether the influence of childhood trauma changes with the stage of depressive illness, especially its associations with the inflammatory system. This study provides new evidence by comparing adolescents with first-episode MDD and recurrent MDD.

Regarding clinical features, this study found that childhood trauma was positively correlated with more severity of depression, suicidal ideation, and alexithymia in adolescents with MDD. These associations were consistently observed in both first-episode and recurrent patients, supporting the notion that childhood trauma exerts a long-term detrimental effect on mental health. Firstly, Evidence from prior research shows that a history of childhood trauma is associated with greater depression severity among patients with depressive disorders (36, 37). For example, a cross-sectional study by Chen et al. (36) reported that childhood trauma has a cumulative effect on depressive symptoms in college students. During the occurrence and development of depression, the negative cognitive patterns and perfectionist tendencies formed by childhood trauma experiences may play an important role in promoting and maintaining it (38, 39). Another study suggested that emotional abuse and neglect may impair anticipatory pleasure and cognitive reappraisal by altering the structure of the ventral striatum and cingulate cortex, which could be an important neural mechanism through which childhood trauma contributes to more severe depression symptoms (40).

Secondly, childhood trauma is strongly associated with an elevated risk of suicidal ideation, and this association which has been consistently demonstrated in the literature (41, 42). From a psychological perspective, individuals with childhood trauma frequently experience more intense feelings of pain and despair, along with heightened irritability and impulsivity, which substantially elevates their suicide risk (43, 44). The study by Wang et al. (45) revealed that, childhood emotional abuse may indirectly increase suicidal ideation by triggering rumination and experiential avoidance, thus identifying emotional dysregulation as a critical mediating mechanism. In terms of biological mechanisms, childhood trauma may result in dysregulation of the hypothalamic-pituitary-adrenal (HPA) axis. This dysregulation is often manifested as a blunted cortisol secretion response to stress, resulting in lower cortisol levels during stress and thereby increasing suicide risk (46, 47).

Finally, Early exposure to childhood trauma may foster an insecure environment, adversely affecting the development of emotional perception and processing in adolescents, contributing to the emergence of alexithymia, which features difficulty with emotional identification, description, and expression (8). For example, a network analysis study in China observed a significant link between exposure to childhood trauma and the occurrence of alexithymia in adolescents with MDD (48). Similarly, a previous cross-sectional study demonstrated that emotional abuse and neglect showed a strong connection to alexithymia in patients with MDD (49). Therefore, ongoing clinical attention to childhood trauma history should be given during the long-term treatment and follow-up of adolescents with MDD.

Notably, this study also found that adolescents with MDD had significantly higher plasma levels of several inflammatory cytokines (IL-1β, IL-6, IL-10, IL-17A, TNF-α) compared to HCs, a result supported by previous domestic and international research (50–52). For example, Becerril et al. (51) found that the peripheral IL-17A levels in adolescents with MDD were significantly higher than those in HCs. And this elevated inflammatory state showed a strong association with the patients’ childhood traumatic experiences. A case-control study reported that, compared to HCs, untreated adolescents with MDD not only had more severe childhood trauma and higher plasma IL-6 levels but also exhibited a significant positive correlation between these two factors (53). The close correlation between childhood trauma and high levels of inflammatory cytokines in patients with MDD may involve complex mechanisms. Firstly, childhood trauma has been shown to be significantly associated with elevated serum HMGB1 levels in adolescents with depression (54). HMGB1 is a key upstream mediator that activates the TLR4/NF-κB signaling pathway and drives transcription of pro-inflammatory cytokines including IL-1β, IL-6, and TNF-α (55). This mechanism is supported by clinical evidence of HMGB1 nuclear-to-cytoplasmic translocation observed in depressed patients (56). Secondly, childhood trauma may affect the DNA methylation status of the FKBP5 gene in peripheral blood, leading to the activation of the major immune regulatory factor NF-κB, which promotes a pro-inflammatory state and contributes to the development of depressive disorder (57, 58). Thirdly, evidence implicateed childhood trauma in processes such as heightened lipopolysaccharide sensitivity and microglial priming, potentially underlying a heightened vulnerability to comorbid mental and somatic illnesses (59, 60). Furthermore, employing the maternal separation model of early-life adversity, animal studies have shown a significant correlation between decreased prefrontal cortical parvalbumin levels and elevated plasma inflammatory cytokines (including IL-1β and IL-6) in adolescent rats (61).

Significantly, our study found a disease-stage specific association between childhood trauma and levels of inflammatory cytokines. While childhood trauma was correlated with and could independently predict levels of IL-1β, IL-6, IL-10, and IL-17A in first-episode patients, all inflammatory associations disappeared in recurrent patients. This first-episode specificity may reflect either a state-dependent inflammatory response during acute depression or a transient biological phenomenon specific to illness onset. These possibilities warrant longitudinal investigation. However, we cannot exclude that medication effects rather than illness chronicity explain the absent correlations in recurrent patients, as 67% were medicated versus 0% in the first-episode group. A prospective study found that plasma levels of IL-6 and IL-1β dropped significantly from baseline in adolescents with first-episode MDD following 6 to 8 weeks of fluoxetine treatment (62). A meta-analysis has demonstrated that antidepressant treatment may lead to a significant reduction in the levels of inflammatory cytokines (including IL-6, TNF-α, and IL-10) among depression patients (63). Thus, drug exposure may confound comparisons between first-episode and recurrent patients.

In addition, We also found that the plasma levels of IL-1 β and IL-8 in first-episode adolescents with MDD may be positively correlated with alexithymia. Our findings on IL-8 are similar to those of Uher et al. (64), who reported elevated cerebrospinal fluid IL-8 levels may be linked to alexithymia in non-inflammatory neurological disorders. In contrast, the positive correlation we observed between IL-1β and alexithymia diverges from previous reports. Mandarelli et al. (65) reported no significant difference in serum IL-1β levels between alexithymic and non-alexithymic subjects undergoing upper endoscopy, while Peters et al. (66) found that impaired facial recognition in adolescents was correlated with IL-1β levels. Due to the cross-sectional design, this study did not include any intervention. Future research with larger samples and prospective interventional designs is warranted to further validate the causal relationship between these variables.

There were some limitations to this study. First, although childhood trauma temporally precedes depression, our cross-sectional design limits causal inference. We could not dynamically observe the evolution of inflammatory and clinical symptoms post-trauma, nor fully rule out confounding by factors like genetic susceptibility or adverse environments. Hence, it remains unclear whether the observed first-episode specificity reflects a genuine temporal progression or is attributable to selection bias. Second, the modest sample size may have limited statistical power, particularly for subgroup analyses. The first-episode subgroup (n = 75) may be underpowered for multiple regression analyses, especially when examining cytokine associations with modest effect sizes, thereby increasing the risk of both Type I and Type II errors. Third, childhood trauma was assessed via retrospective self-report, a method susceptible to recall bias, mood-congruent memory effects in currently depressed adolescents, and social desirability bias, all of which may inflate its observed associations with current symptoms. Fourth, medication status was confounded with illness stage (0% vs. 67% medicated), precluding disentanglement of pharmacological effects from disease chronicity; other confounders (lifestyle, comorbidities, social support) were also unmeasured. Additionally, the regression models adjusted only for illness duration and BMI, without controlling for pubertal stage, recent life stressors, or socioeconomic status. Future studies should employ large-scale longitudinal designs to track patients from first onset to remission, clarifying the temporal dynamics among trauma, inflammation, and symptoms. Integrating neuroimaging and molecular techniques will be key to elucidating the mechanisms underlying the observed stage-specific associations.

## 5 Conclusion

In summary, this study found that childhood trauma is prevalent among adolescents with MDD and is closely associated with more severity of depression, suicidal ideation, and alexithymia. Notably, its association with inflammatory cytokines exhibits clear first-episode specificity. This first-episode specificity may reflect a state-dependent inflammatory response during acute depression, a transient biological phenomenon at illness onset, or confounding by antidepressant treatment. These possibilities require longitudinal studies to disentangle. Therefore, in clinical practice, childhood trauma should be routinely assessed in adolescents with MDD. For those with a history of trauma, active interventions should be implemented, aiming not only to alleviate clinical symptoms but also to mitigate inflammation, thereby potentially improving long-term prognosis.

## Data Availability

The raw data supporting the conclusions of this article will be made available by the authors, without undue reservation.
