# Supplementary material for: Associations of childhood trauma with clinical features and inflammatory cytokines in adolescents with first-episode and recurrent major depressive disorder
Source: Front Immunol. 2026 Mar 3;17:1787595. doi: 10.3389/fimmu.2026.1787595 (PMC12991973; doi:10.3389/fimmu.2026.1787595)

Supplementary Material

1 Supplementary Table

**Supplementary Table 1. Significant correlations (*P* < 0.05) of CTQ scores with other variables in first-episode MDD.**

| **Variables** | **First-episode MDD (*n* = 75)** | |
| --- | --- | --- |
|  | ***r*** | ***P*** |
| Duration of illness (months) | 0.241 | **0.037** |
| CES-D scores | 0.396 | **<0.001*** |
| PANSI scores | 0.519 | **<0.001*** |
| TAS-20 scores | 0.454 | **<0.001*** |
| Log IL-1β (ng/L) | 0.264 | **0.022** |
| Log IL-6 (ng/L) | 0.228 | **0.049** |
| Log IL-10 (ng/L) | 0.253 | **0.029** |
| Log IL-17A (ng/L) | 0.251 | **0.030** |
| CES-D, center for the epidemiological studies depression scale; PANSI, positive and negative suicidal ideation scale; TAS-20, 20-item toronto alexithymia scale; CTQ, childhood trauma questionnaire; IL, interleukin. **P* < 0.05/45 = 0.001 (Bonferroni correction). Bolded P value: < 0.05. | | |

3 Supplementary Figures

Fig. 1.


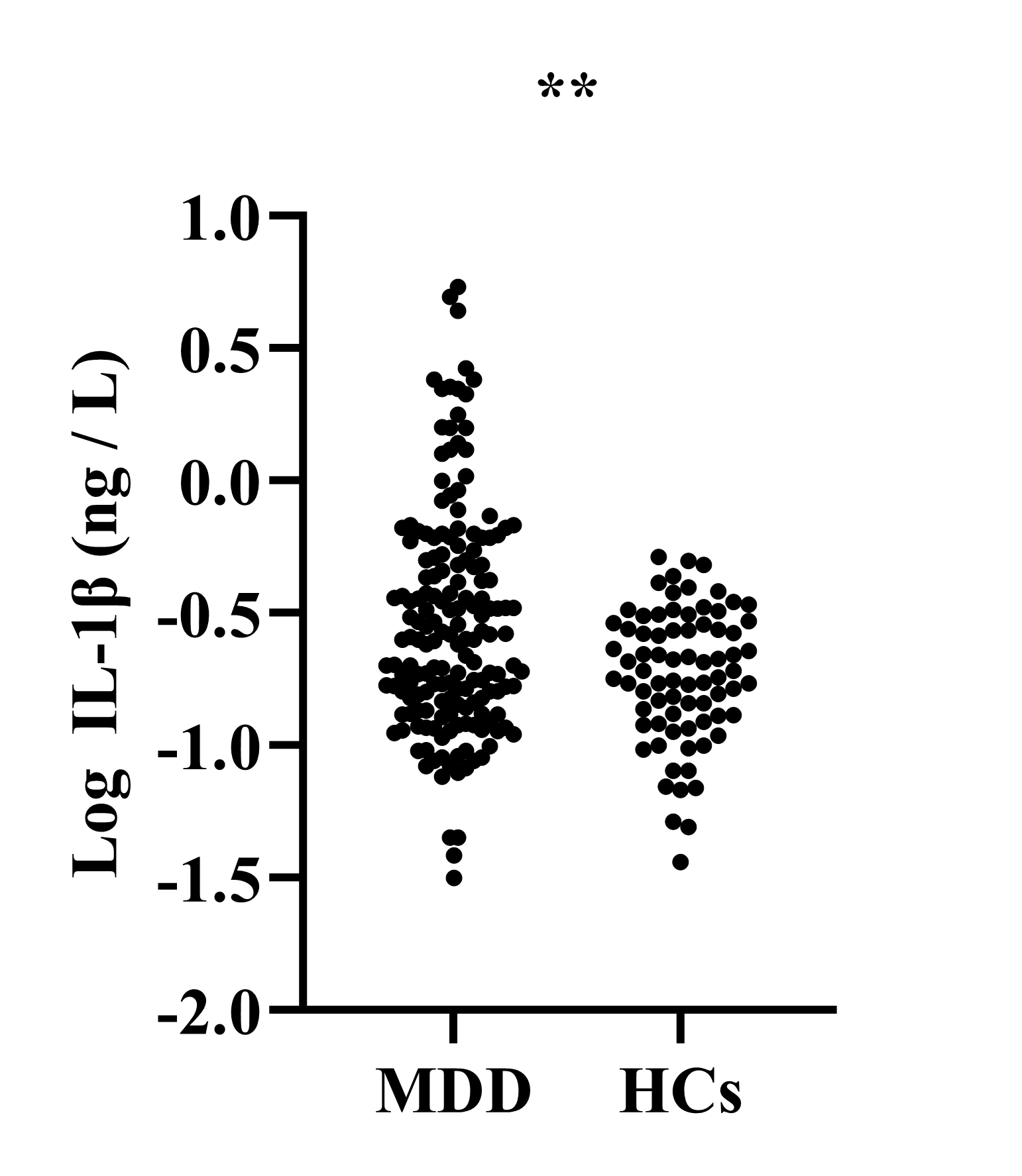

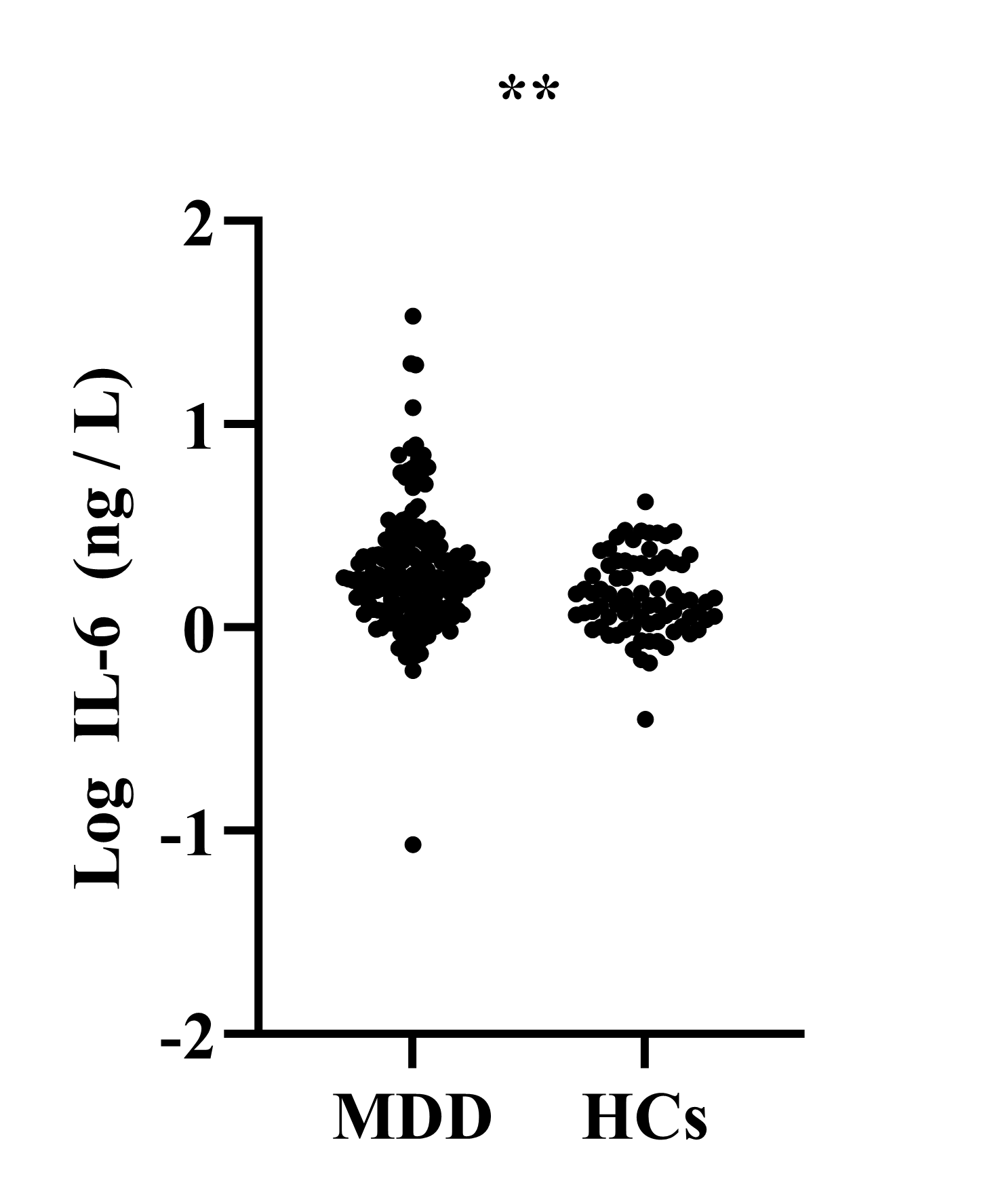


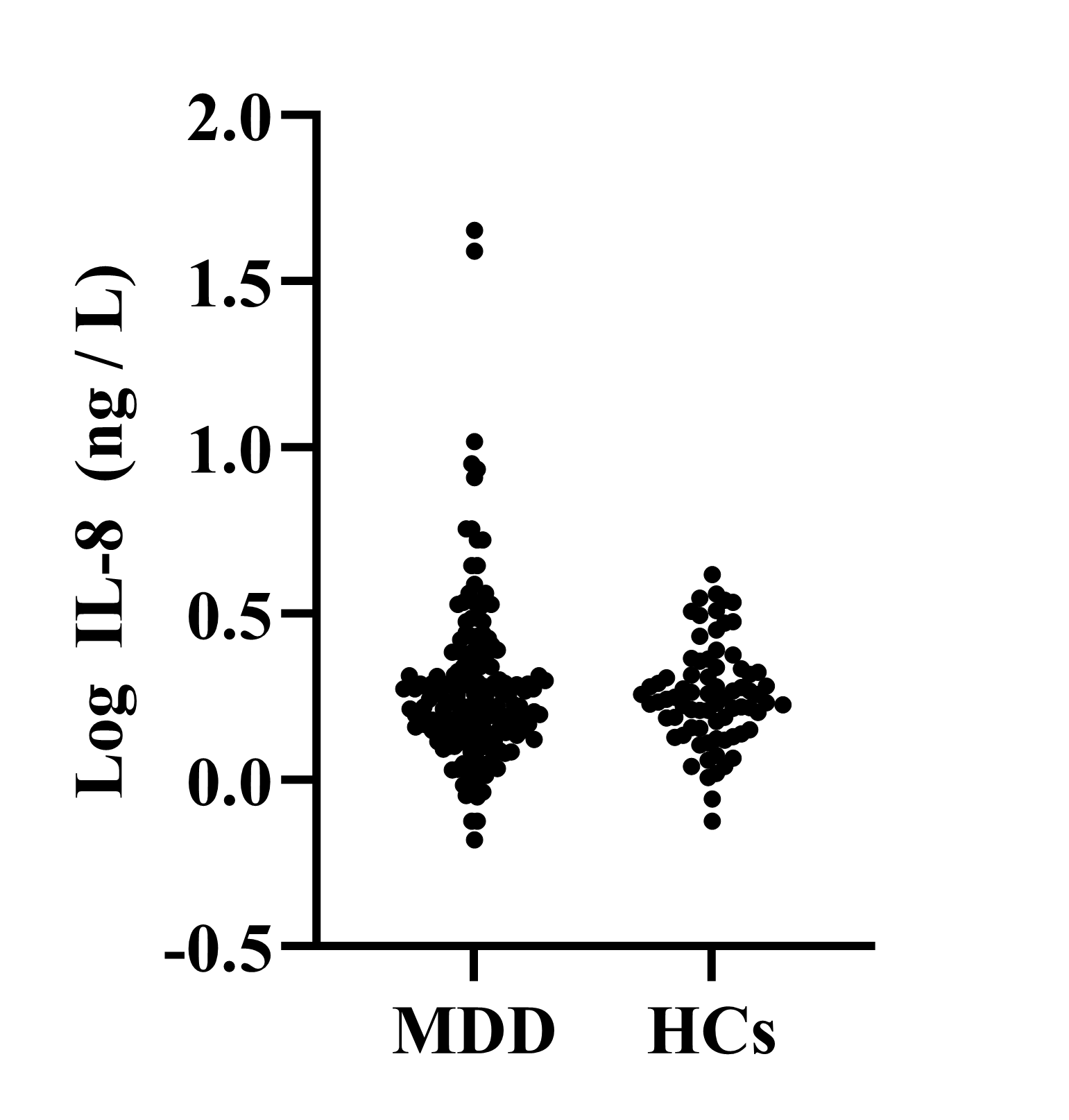

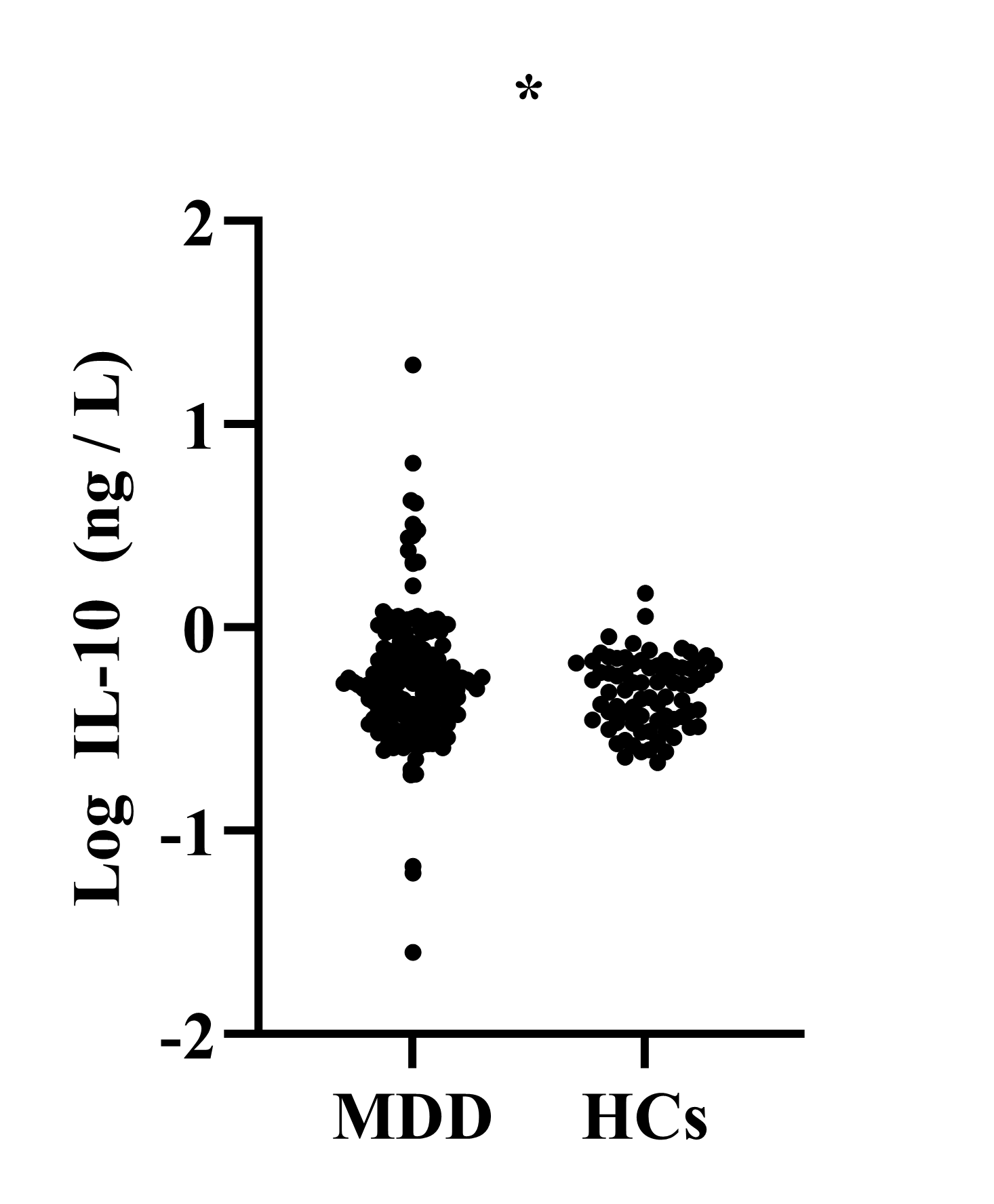


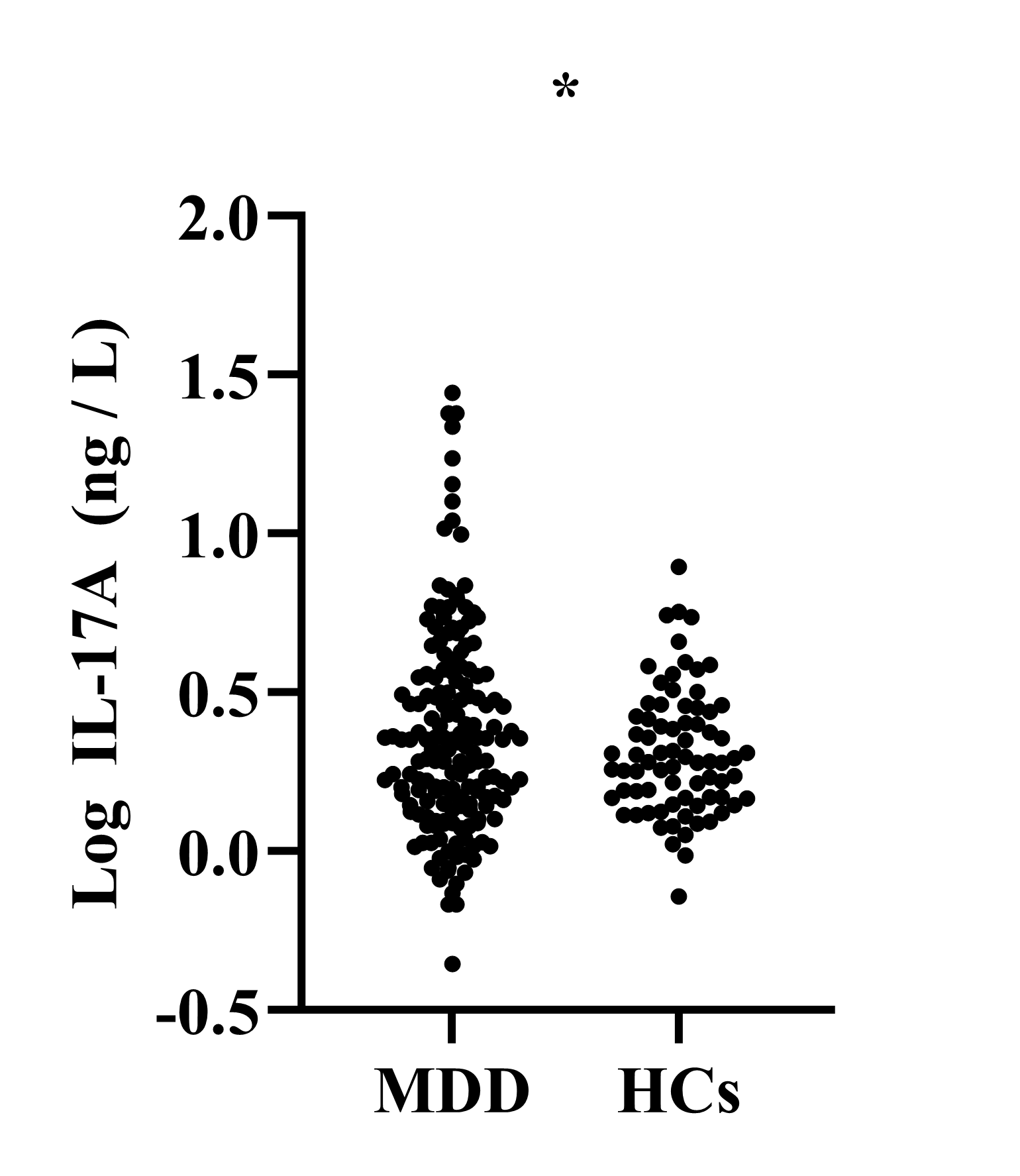

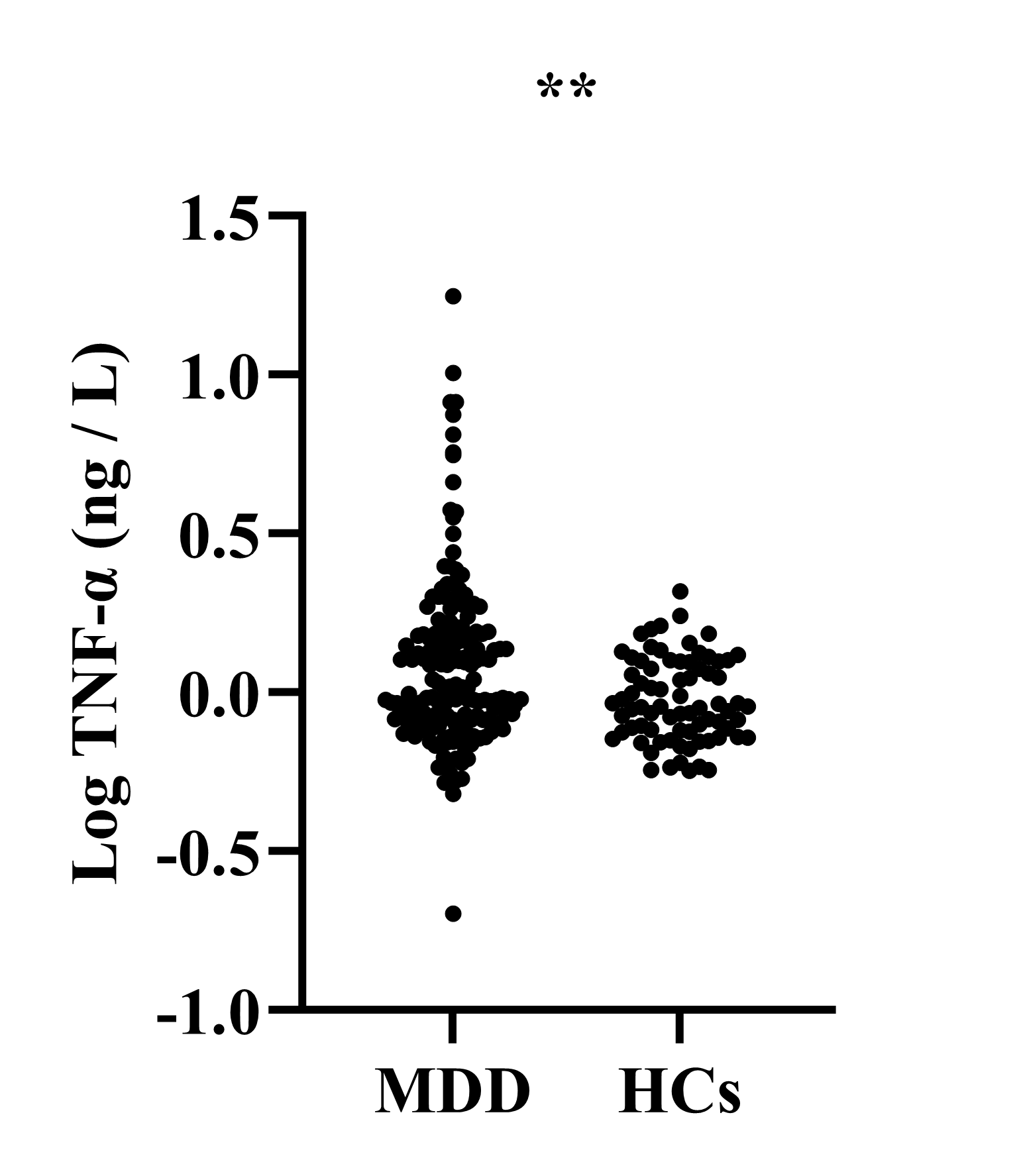


Fig. 2.


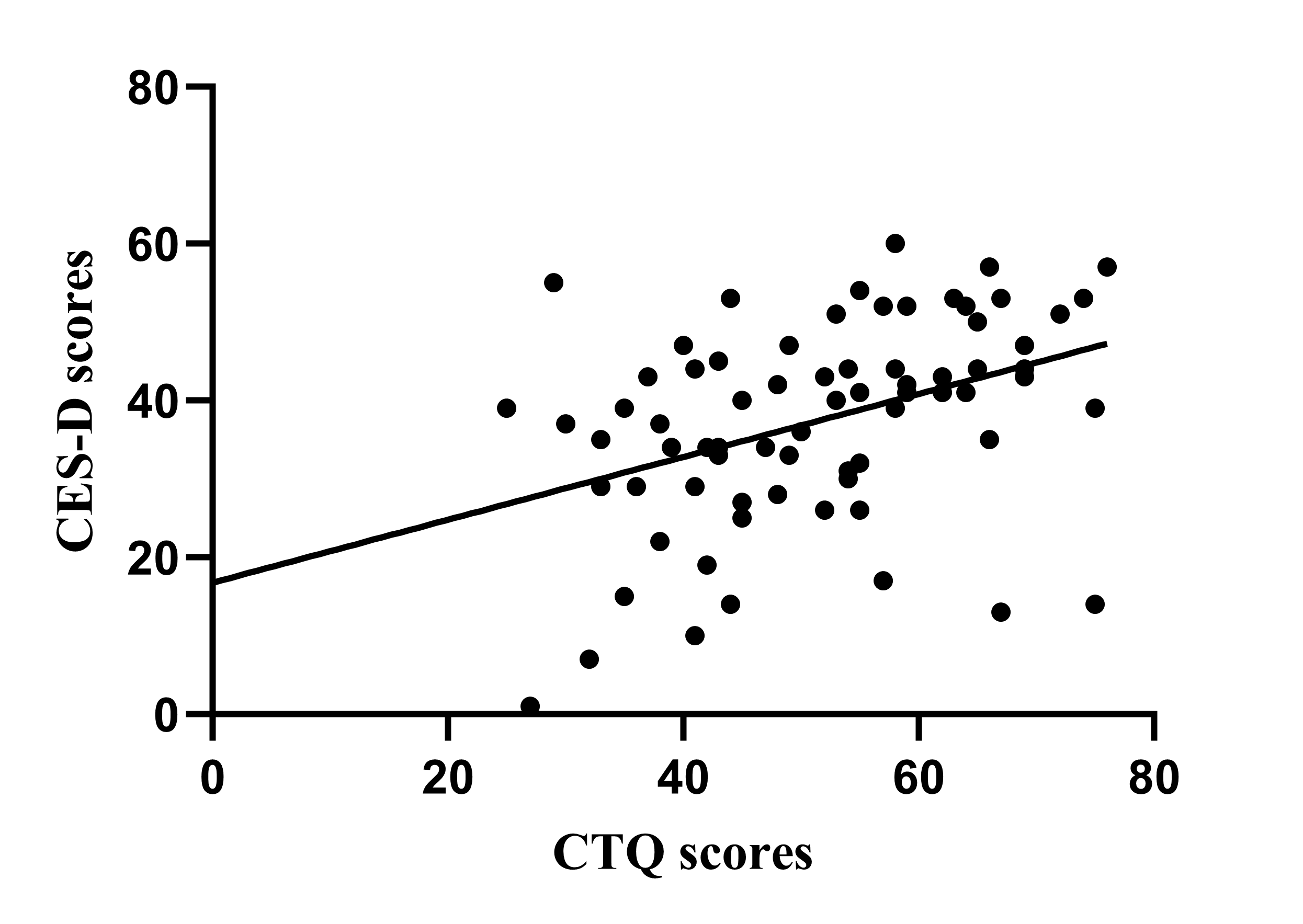

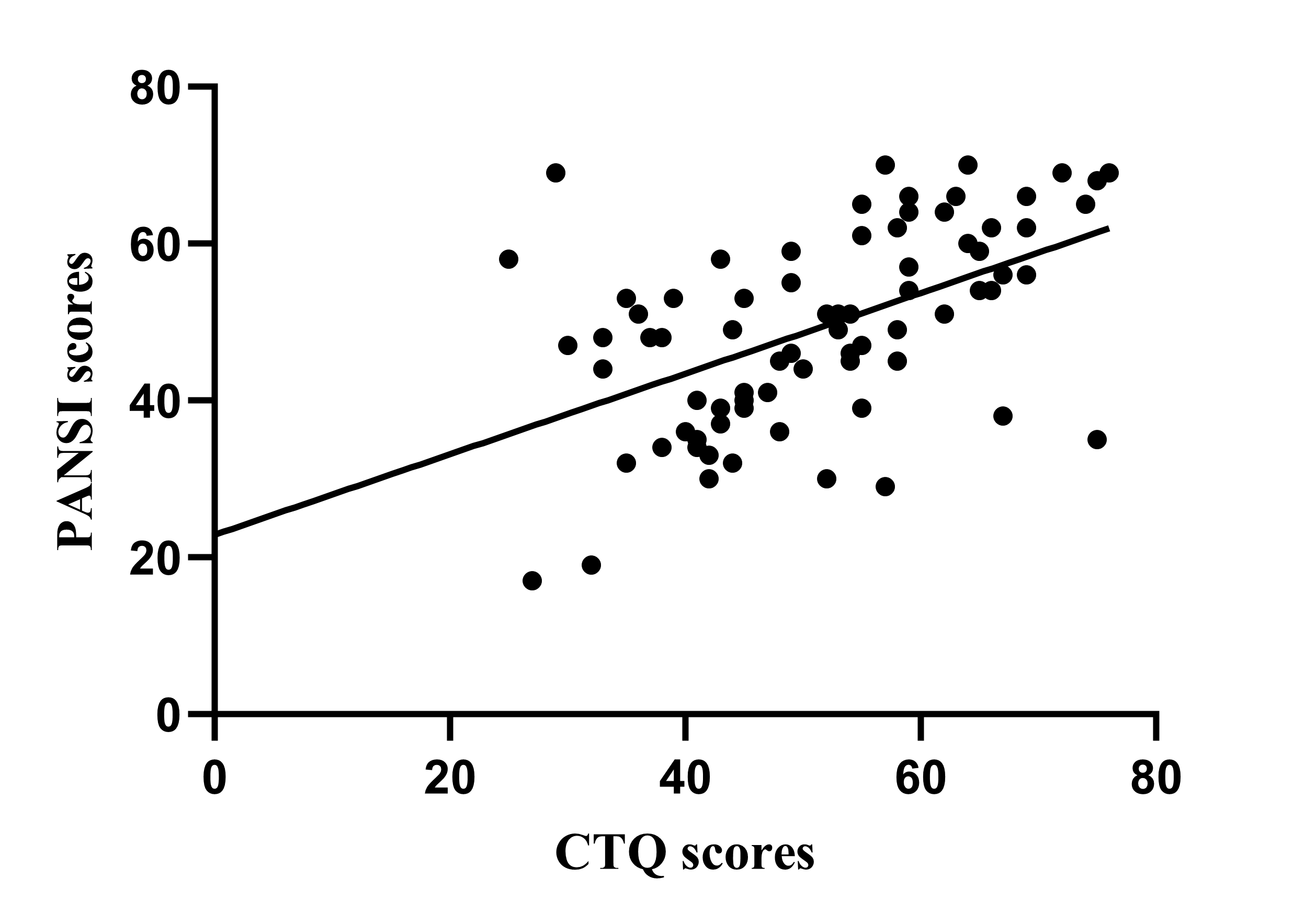

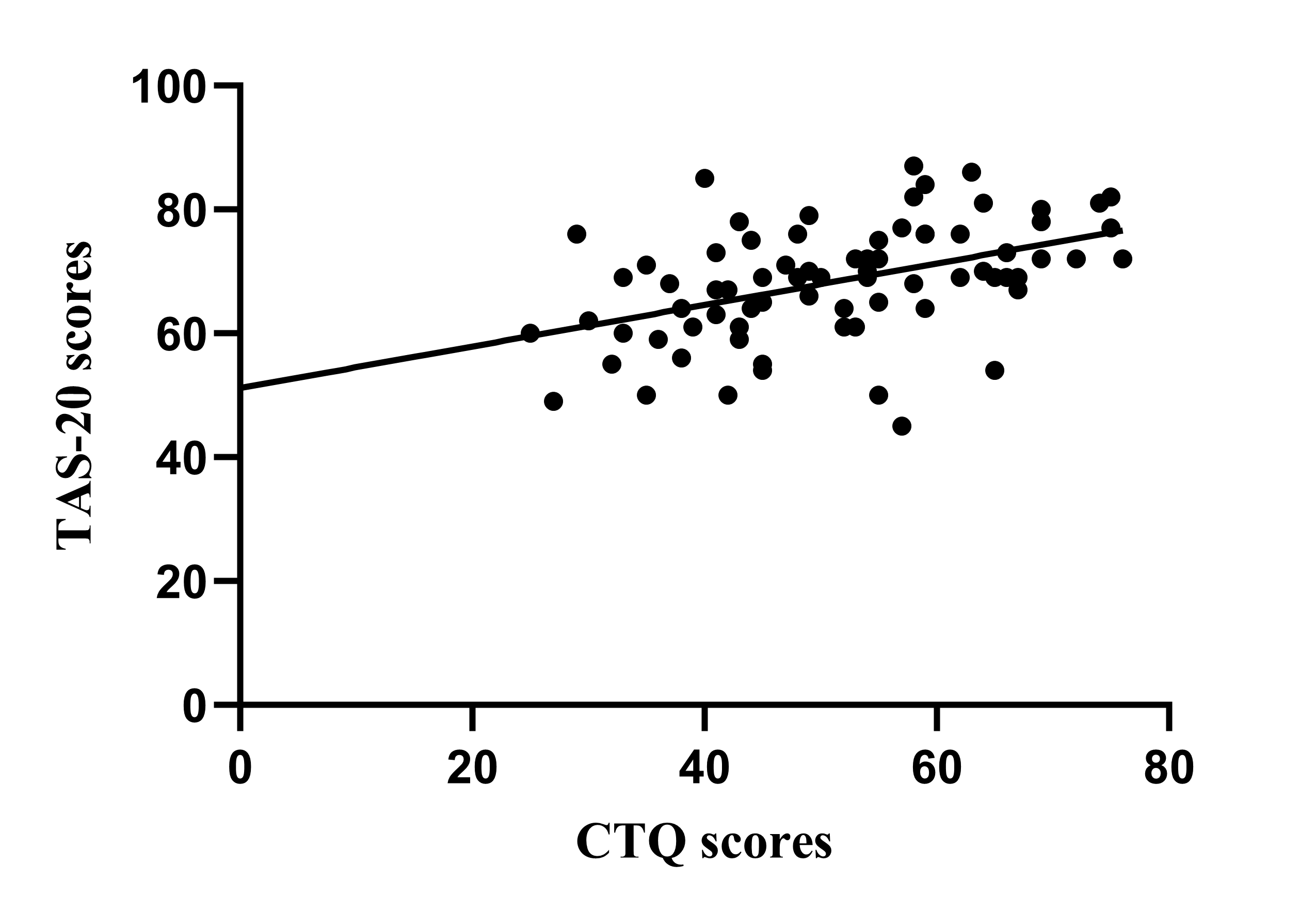

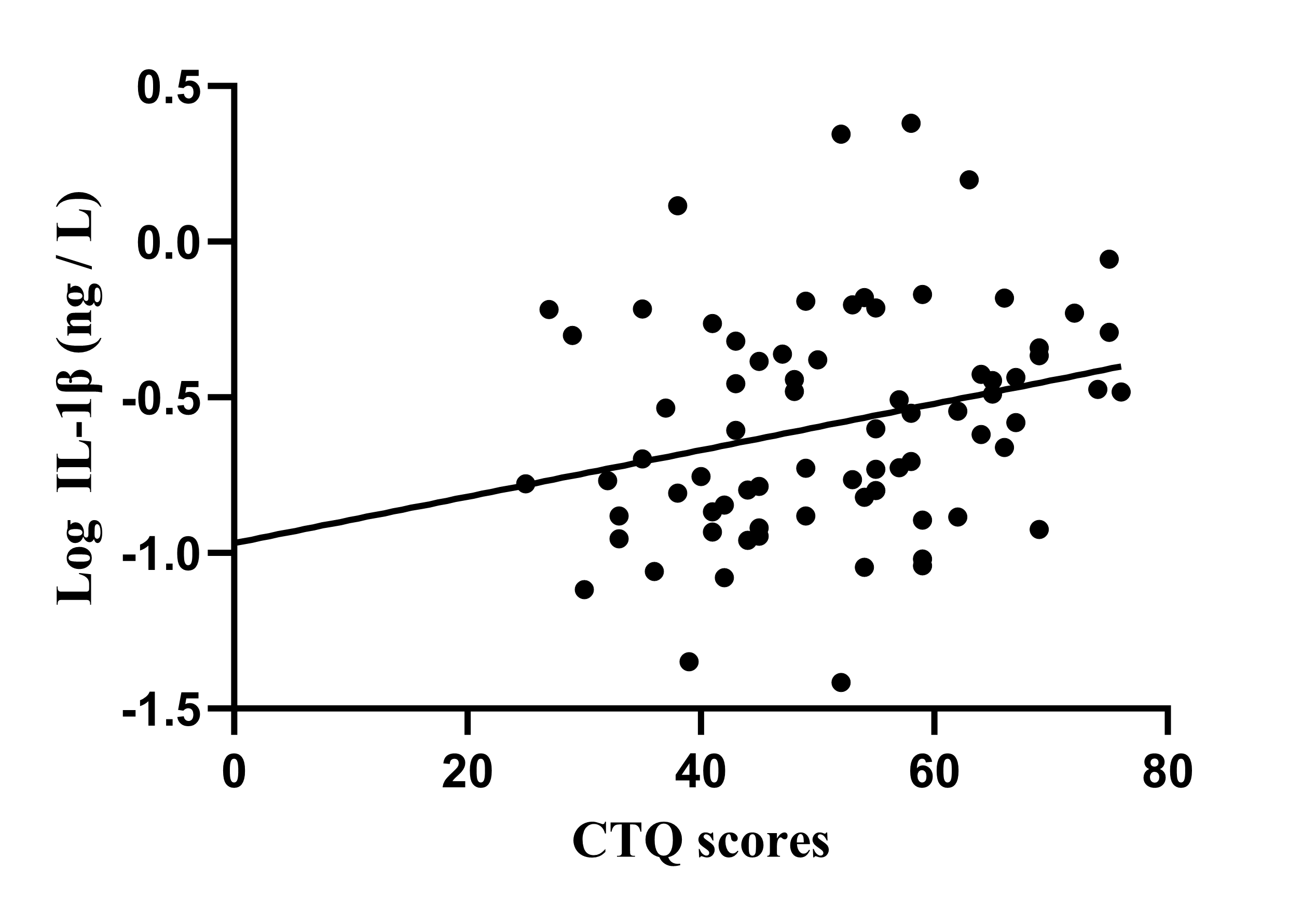

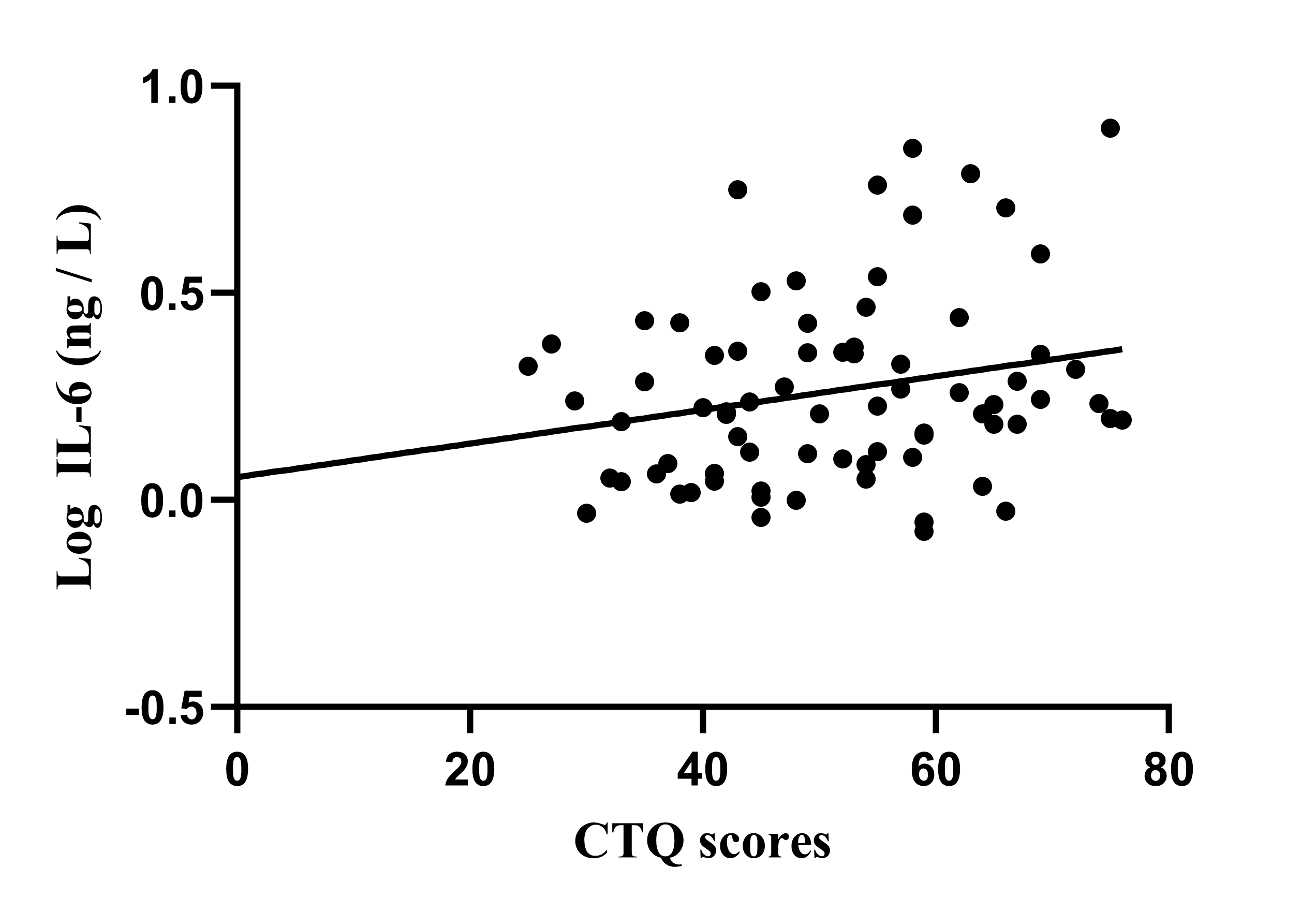

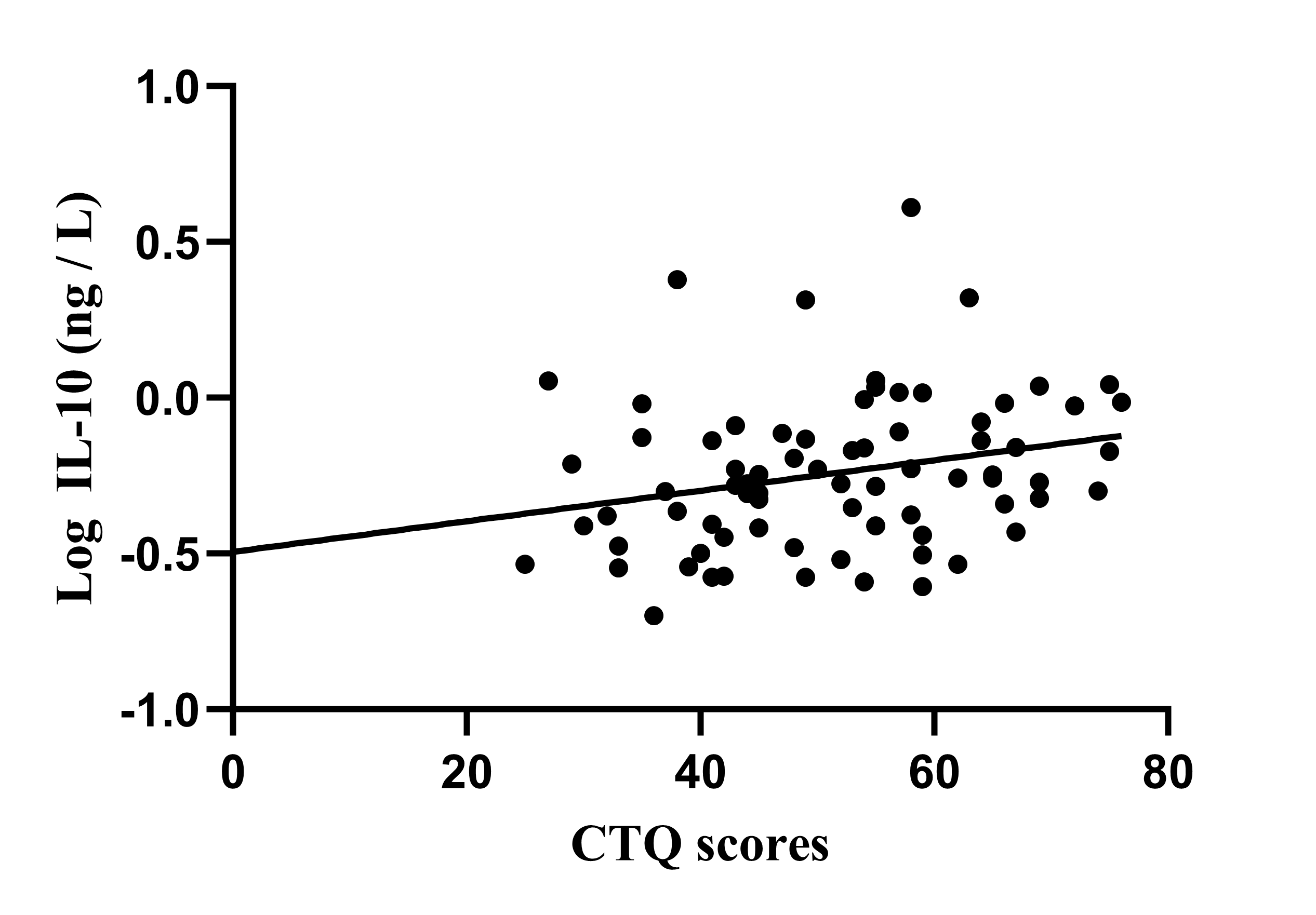

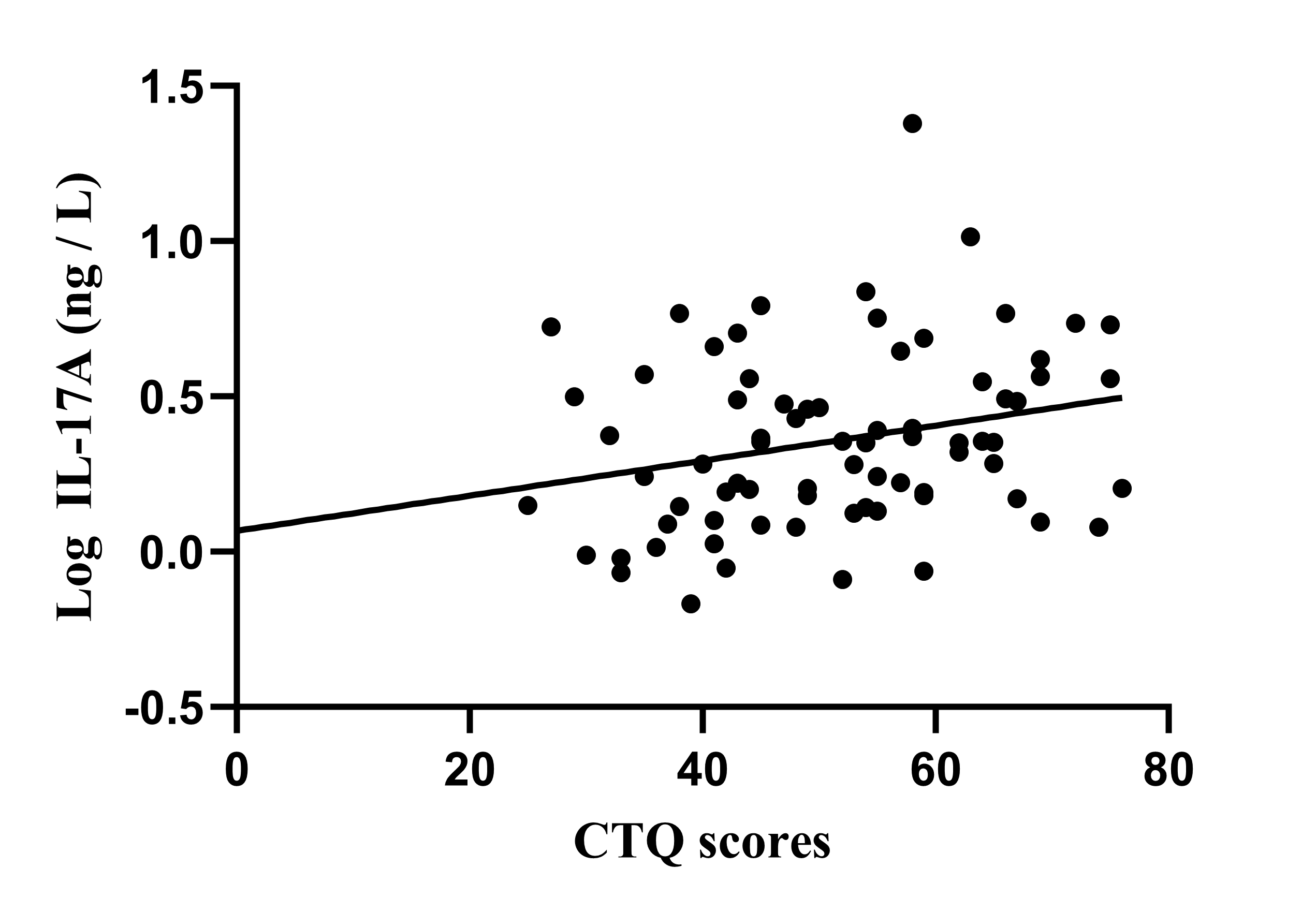


Fig. 3.


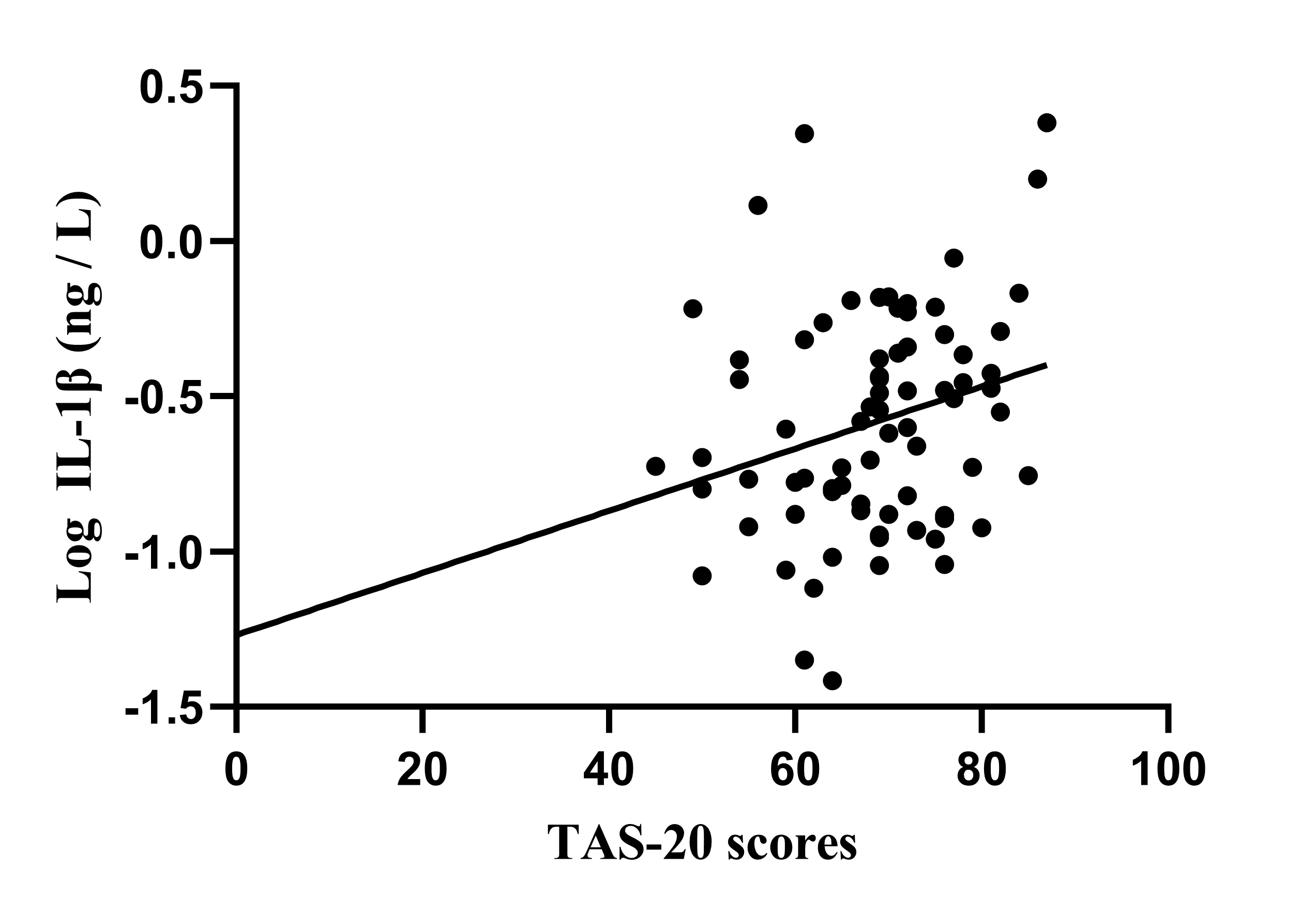

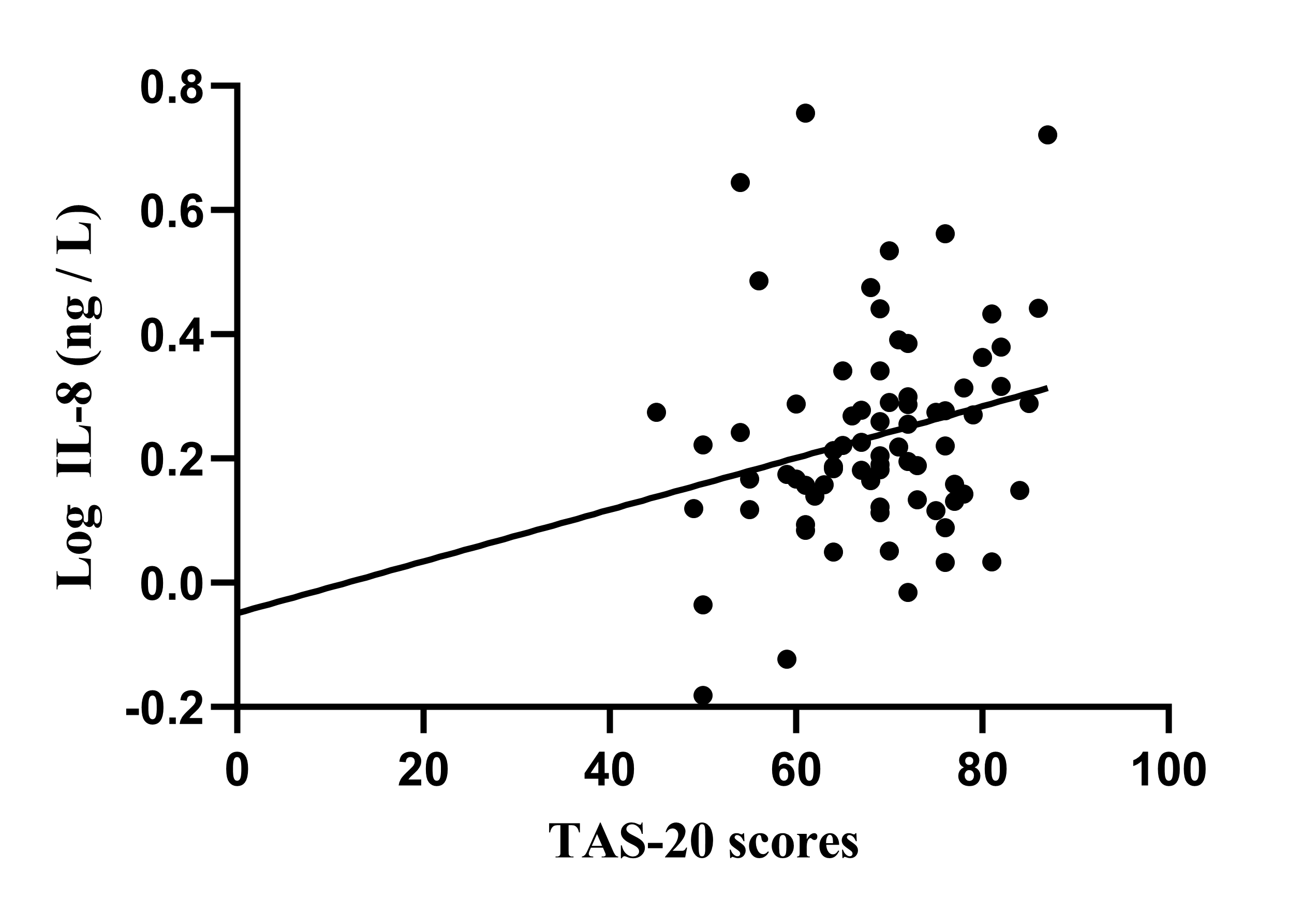

Supplement: Supplementary file 1 [file DataSheet1.docx]
